# Professional Development of Behavior Analysts in Europe: A Snapshot for 21 Countries

**DOI:** 10.1007/s40617-022-00754-0

**Published:** 2022-11-10

**Authors:** Mickey Keenan, Karola Dillenburger, Marie-Hélène Konrad, Natacha Debetencourt, Rea Vuksan, Lefki Kourea, Karel Pancocha, Sheri Kingsdorf, Henriette Juul Brandtberg, Nursel Ozkan, Helene Abdelnour, Magali Da Costa-Meranda, Steffi Schuldt, Robert Mellon, Alexandra Herman, Alan Tennyson, Shiri Ayvazo, Paolo Moderato, Natasha Attard, Jacqueline Schenk, Anna Budzinska, Javier Virues-Ortega, Lise Roll-Pettersson, Dag Strömberg, Silja Wirth, Charlotte Escané, Erika Glaus-Stuessi, Alla Moskalets, Stephen Gallagher

**Affiliations:** 1 Ulster University, Coleraine, UK; 2 Queen’s University Belfast, Belfast, UK; 3 PCM, Vienna, Austria; 4 Univeristy of Nicosia, Nicosia, Cyprus; 5 Masaryk University, Brno, Czech Republic; 6 Danish Association for Behaviour Analysis, Copenhagen, Denmark; 7 ONPAC, Organisation Nationale des Professions de l’Analyse du Comportement, La Gaude, France; 8 Panteion University of Social and Political Sciences, Athina, Greece; 9 Association for Behaviour Analysis Hungary, Budapest, Hungary; 10 Irish Association for Behaviour Analysis, Dublin, Ireland; 11 Kinneret Academic College, David Yellin Academic College, Jerusalem, Israel; 12 IESCUM, Parma, Italy; 13 Erasmus University Rotterdam, Rotterdam, Netherlands; 14 Institute for Child Development (IWRD), Gdansk, Poland; 15 ABA Spain, Barcelona, Spain; 16 University of Stockholm, Stockholm, Sweden; 17 ABA Switzerland, Gossau, Switzerland; 18 Ukrainian Association of Behavior Analysts, Mariupol, Ukraine

**Keywords:** professional recognition, Europe, behavior analysis, Behavior Analyst Certification Board

## Abstract

Behavior analysts are not recognized or regulated as a distinct profession in Europe. For the most part, European behavior analysts adhered to the standards set by the U.S.-based Behavior Analyst Certification Board (BACB). However, the BACB certification has not been recognized officially in any European jurisdiction. The recent decision by the BACB to discontinue eligibility of non-U.S. residents to apply for the BCBA exam by the end of 2022 has brought the issue of professional regulation outside of the United States into sharp focus. This article offers a snapshot in time of professional recognition of behavior analysts in 21 European countries. It stems from the Erasmus+ funded EuroBA project and its Professional Advisory Group (PAG). The EuroBA project aims to develop common standards and competences for behavior analysts to facilitate national regulation and mutual recognition across Europe.

The global recognition of the professional status of behavior analysis is uneven across continents. In Europe, for example, it is not recognized and practice is largely unregulated. Without professional regulation consumer protection is not guaranteed because there is no agreed definition of the parameters of scope and competence. The result is that service users have limited access to well-trained behavior analysts. In addition, there is restricted cross-border mobility for professionals, and most important, consumer safety is compromised at significant cost (Buescher et al., 2014). The purpose of this article is to give an overview of the current status of the progress in the professionalization of behavior analysis in Europe. We also describe a recent project that aims to facilitate this process.

Professional recognition of behavior analysts in the United States has a long history (Johnston et al., 2017). In fact, all 50 states now recognize behavior analyst certifications, registrations, and/or have licensing laws for behavior analysts (Behavior Analyst Certification Board [BACB], 2022). For the most part, European behavior analysts used the standards set by the U.S.-based BACB (2019) with the aim of becoming board certified behavior analysts (BCBA) or registered behavior technicians (RBT; Hughes & Shook, 2007; Kelly et al., 2019). However, for legal reasons, no foreign qualification can automatically be recognized in another country, neither in Europe (European Commission, 2022; for exceptions across EU member states, see Council of Europe, 1999) nor in the United States (U.S. Department of Education, 2022).

The full realization of this state of affairs was one of the key factors that prompted the BACB to discontinue eligibility of non-U.S./Canada residents to sit for the BCBA exam by the end of 2022.^1^ The accreditation in the United States by the National Commission for Certifying Agencies (NCCA) meant that BACB certification programs “have been written into hundreds of laws and funding policies . . . [and regulators in other countries] may be disinclined to recognize standards that are not necessarily specific to their country and its practices” (BACB, 2019). Of course, it stands to reason that a foreign qualification can only be recognized if it exists as a regulated profession in both the country of origin and the host country (Kelly & Trifyllis, 2022).

The lack of professional recognition of behavior analysts in Europe and, as a result, the lack of regulation of their professional practice, means that anyone can claim to have the expertise to deliver behavior analysis-based services, regardless of their level of training, skills, ethical stance, or area of practice (Heward et al., 2022). It is regrettable that a certain amount of unprofessional conduct has crept into service delivery in some places (Keenan et al., 2010; cf. Sallows, 2000) and a credible rejection of the use of aversive procedures in early records (Lovaas, 1987; European Association of Behaviour Analysis [EABA], 2022; Dillenburger & Keenan, 1994; Keenan & Dillenburger, 2020; Leaf et al., 2018; Odom, 2021) has meant that condemnation of ABA has gained traction in other quarters (Milton, 2012). This is unfortunate, in particular because the science of behavior analysis has a long history of rejecting the use of aversives. In fact, B. F. Skinner received the Humanist of the Year Award in 1972 from the American Humanist Society in part for his detailed and progressive ideas of how to replace the kind of aversive control that is commonplace in society with systematic positive reinforcement contingencies (Skinner, 1948/1976, 1971, 1972); Sidman (1989) exposed the adverse fallout of the use of coercive control within society in general.

A further complication is that some branded programs that are based on applied behavior analysis (ABA) are promoted without accurate reference to their scientific heritage. This has left service users confused and unable to differentiate between ABA and specific kinds of services. For example, some advocates of positive behavior support (PBS), a clinical approach to challenging behavior that is grounded in ABA (National Institute of Clinical Excellence [NICE], 2015; Sailor et al., 2009), recently distanced themselves publicly from ABA (British Institute for Learning Disability [BILD], 2022; National Autistic Society [NAS], 2022). What is particularly disconcerting about this is the fact that PBS clearly falls within the scope of practice of behavior analysts (Heward et al., 2022) because practitioners cannot claim to apply PBS properly without using the principles and practices associated with behavior analysis (Sailor et al., 2009). In fact, PBS falls within the scope of competence for many behavior analysts (Brodhead et al., 2018). On the other hand, it would not be the case that someone who is trained solely in PBS could claim the wide scope of practice or the certification of a behavior analyst (e.g., Heward et al., 2022, cite 350 different areas of practice of behavior analysis, PBS being one of these). Of course, there is significant value of PBS in that it “has put a spotlight on previously unregulated technologies in behavior analysis and given strength to the non-aversive movement” (Fielding et al., 2013, p. 89). However, the failure to differentiate between scope of practice and scope of competence in some contexts where ABA is not established has led to the promotion in government policies of one service model at the expense of disseminating accurate information about the whole discipline of ABA (Department of Health, England, 2014).

There is no doubt that professional regulation of behavior analysts is crucially important to address these and other issues, not least to ensure service users have access to properly trained and regulated behavior analysts who offer best practice (Blakemore, 2021) and to improve cross-border mobility, but to also protect service users from harm (McGill & Robinson, 2021; Taylor et al., 2019). It is overdue in Europe. In fact, professional recognition and regulation will bring behavior analysts in line with other clinical, allied health, and education professionals to ensure that incidents of malpractice and abuse can be addressed quickly and appropriately (Dillenburger et al., 2014).

In keeping with the philosophy of the European Commission concerning transparency, mutual trust, and high educational standards, the European Qualifications Frameworks (EQF) evolved as a means to define professional standards through development of a common reference frameworks thereby supporting not only good practice but also cross-border mobility. In general, in order to achieve mutual recognition, a profession needs to be recognized and regulated with a country, before mutual recognition agreements can be negotiated across Europe. The European Parliament and the Council of the European Union (2013) clarify the necessary procedures. Mutual recognition across Europe has been agreed only for a limited number of professions, including doctors, nurses, dentists, veterinary surgeons, midwives, pharmacists, and architects. Such recognition is multilateral and based on harmonized minimum training requirements, a general system for the recognition of evidence of training, and recognition of professional experience.

For professions that are not eligible for automatic recognition, qualifications may be recognized under Article 45 of the Treaty on the Functioning of the European Union (TFEU; free movement of workers) or under Article 53 of the TFEU (freedom of establishment). “In these cases, the competent authority of the host Member State must compare your training with its national training by taking into account your professional experience and any further training. If the training corresponds only in part, it may ask you to make up for these differences, for example by taking a test, doing a traineeship or an additional training course, depending on the national rules” (European Union, 2020, p. 26). As a result, it is important to establish comparable standards and professional recognition for behavior analysts across Europe.

Although some European countries had started to work towards professional recognition of behavior analysts in their own jurisdictions prior to BACB’s decision, others mistakenly believed that the U.S.-based certification would provide sufficient professional recognition and service user protection for professionals in their own countries. They did not realize that ultimately they could not avoid the labor-intensive process of gaining professional recognition in their own countries. As a result, European countries currently are at different stages in the process of gaining professional recognition and regulation for behavior analysts.

The present article reports the state of affairs for behavior analysts in 21 European countries. The document stems from the EuroBA (European Behavior Analyst) project in collaboration with its Professional Advisory Group (PAG). The EuroBA project is a 3-year project (September 2020–August 2023) funded through the European Commission Erasmus+ program. The project is led by Professor Mickey Keenan (Ulster University, Northern Ireland) and includes nine partners from seven European countries (United Kingdom [UK], Greece, Sweden, Italy, Ireland, Czech Republic, and the Netherlands). The EuroBA project was conceived with the realization in mind, that common standards and curricula would have the potential to ease the administrative burden on countries aiming to achieve professional recognition of behavior analysts in their jurisdiction and, at the same time, eventually facilitate mutual recognition across Europe. The EuroBA project is producing six *intellectual outputs* (IO):

1. Map the profession of behavior analysts onto the European Qualifications Framework (EQF).
2. Describe progress in the six partner countries based on their National Qualifications Frameworks (NQF) and show how these maps onto the EQF.
3. Produce a glossary of terms that facilitates a common language among behavior analysts.
4. Outline key competences for EuroBA-T (technical entry predegree level) and offer a guide for course developers.
5. Outline key competences for EuroBA-M (Masters level) and offer a guide for course developers.
6. Provide an online multimedia entry, predegree level, technician course (using programmed instruction; Twyman, 2020) that is adapted and translated into partner languages.

The EuroBA project will be completed at the end of August 2023 and the IOs will be freely available to any country aiming to progress professional recognition in their jurisdiction and targeting mutual recognition across Europe and further afield (http://euroba.org).

The Professional Advisory Group (PAG) was set up to ensure that European and allied countries who are not partners of the funded EuroBA project have an input to the intellectual outputs that are developed in the EuroBA project. At the time of writing, 15 additional countries were represented on the PAG. Membership of PAG is open to all counties and includes one representative per country. Each member of the PAG represents the national organization/s that lead the developments for seeking professional recognition of behavior analysts in their home country. In countries where such an organization does not yet exist, or is in an early planning stage, a designated behavior analyst is a member of the PAG.

This document by no means offers the complete picture across Europe. It simply presents a snapshot in time. Countries not represented in the present article who are keen to join the PAG are welcome. The descriptions of the state of play of the 21 countries in this article are presented in alphabetical order. It will be seen that countries are at different stages of development of the dissemination of ABA, the foundation of a national association, training opportunities, and professional recognition of behavior analysts and, as a result, the descriptions reflect national similarities and differences.

## Austria

The situation in Austria is limited, because behavior analysts are almost nonexistent. Indeed, there are two internationally graduated BCBA psychologists in Austria as of October 6, 2021, who work with individuals with developmental disabilities; one person has completed her MSc in ABA and her PhD at Queen’s University Belfast, on applied aspects of behavior analysis with human–dog dyads.

According to DeSouza and Konrad (2021), the situation in Austria presents several barriers to the practice of behavior analysis. First, the benefits of behavior analysis are not recognized by the Austrian government. This has, among other things, a consequence for interventions with individuals with developmental disabilities because they are not funded by the government (only speech and language therapy, physical therapy, music therapy, and occupational therapy are funded). There are some agencies that offer ABA-based programs for individuals with developmental disabilities, but as there are no academic programs in behavior analysis in Austria (in contrast to some border countries such as Italy, Czech Republic, or Hungary that offer a Verified Course Sequences [VCS] verified by the Association for Behavior Analysis-International [ABAI, 2021]), the number of ABA-based service providers is limited. A professional wishing to train in behavior analysis will have no choice but to train abroad. However, the specific regulations of Austria's Social, Health Care, and Consumer Protection Ministry regarding the practice of a psychologist require a master's degree in psychology as well as a minimum of supervised experience by a clinical psychologist, which together represent another significant barrier to the professional practice of a behavior analyst in Austria.

## Belgium

Belgium only has five BCBAs, including three at the University of Ghent (see BACB Certificant Registry). We lack the subsidies for educational practices that are common for targeting socially appropriate behavior for all people with rights to inclusion in a tolerant society. Thus, in Belgium parents of children on the autism spectrum have become increasingly isolated as they cry out for evidence-based practices for their children with autism.^2^ Training is much needed for good practices among professionals and dissemination at university level of behavior analysis is also needed. There are no VCS course sequences for behavior analysis at university level currently in Belgium. The dissemination of behavior analysis in Belgium is sorely needed for concrete intervention in schools, workplaces, and homes.

## Croatia

Recognition of the field of ABA is just emerging in Croatia. Although Croatia does not have a national association or accreditation body yet, some significant changes have occurred since 2017. The University of Zagreb was approved to provide a one-time VCS for approximately 20 students. Thanks to that opportunity, Croatia went from having no certificants to seven BCBAs and six RBTs according to the BACB Certificant Registry. Before the VCS, Croatia had two more senior certificants who moved to the country from North America in an effort to help disseminate ABA.

Given that the emergence of certificants in the country occurred relatively quickly and recently (with five out of seven BCBAs and all six RBTs qualifying in 1.5 years), Croatia does not have a national association, recurring VCS, or an accreditation organization. Most certificants work in the public sector (e.g., day care, schools, nongovernmental organizations [NGO], public intervention services) with only three working privately. Certificants working in the public sector do not necessarily state openly that they provide ABA-based services, but rather work under their core professions (e.g., special education, early childhood education). In addition, there are some uncertified practitioners who provide services that are neither regulated nor quality controlled. Inconsistent quality of services along with low national awareness of what ABA has contributed to misconceptions and misrepresentations of the field.

In response to their membership in the PAG of the EuroBA project, certificants in Croatia have recently started to meet regularly in an effort to form a national association and possibly a chapter of the ABAI as a first step towards building awareness, increasing consumer protection, and helping to move the field along in Croatia.

## Cyprus

The science of behavior analysis has been growing steadily but slowly in Cyprus over the last 15 years. A small number of behavior analysts who had been trained in the United States and UK returned to Cyprus and started working privately by offering ABA-based services to families and children with disabilities after 2004. In 2011, the Cyprus Behavior Analysis Association (CyABA) was established as a nonprofit organization. The founding members included three BCBA certificants and one person with a PhD in ABA.

The CyABA has focused on promoting the science and practice of behavior analysis as well as establishing the profession of behavior analyst. In October 2017, the Association conducted its first national conference in ABA, attracting great interest from educators, health-care professionals, and parents. Despite efforts to expand the CyABA, the number of people involved in the organization remains small. At the time of this writing, there are six active professionals certified as BCBA and one as BCaBA, who work privately by offering services to families and their children with disabilities. According to data retrieved from the BACB online Certificant Registry on April 28, 2021, there are an additional seven professionals whose status either expired or is inactive.

The future of ABA in Cyprus remains uncertain but promising. Future efforts should include two main goals: (1) creating an VCS (verified by ABAI) with local universities to increase the number of ABA professionals on the island; and (2) pushing forward to establishing the profession of behavior analysis under Cyprus’s existing legislation framework.

## Czech Republic

ABA has been slow to gain momentum in the Czech Republic. Prior to the fall of the Iron Curtain, behavior analysis was virtually unheard of. Behavioral science started to emerge in the late 1990s. From 2015, parents of children with autism built support for ABA by bringing together politicians and national and international academics, organizing the first major ABA conference in Brno, and arranging a parliamentary hearing in 2016 (Gandalovičová, 2016).

The government supported these developments with a five-year plan, subsidizing the development of university-based training in behavior analysis, funding the training of the first 15 Czech behavior analysts through international MScABAs, and setting up the Centre for Applied Behavior Analysis at Masaryk University in Brno (Roll-Pettersson et al., 2020). By 2021, the number of behavior analysts had risen to about 40 practitioners (Kingsdorf & Pančocha, 2020).

During this critical 5-year period, the Czech Society of Applied Behavior Analysis (CSABA) was established in 2016, followed by the foundation of the Working Group for Applied Behavior Analysis of the Czech Medical Society (Kelly et al., 2018). Also in 2016, there was a decree by the Government Committee for Citizens with Disabilities at the Office of the Government of the Czech Republic, which issued an initiative to address the situation of people with autism and their families (Vládní výbor pro zdravotně postižené občany [Government Committee], 2016). It outlined the need to establish a comprehensive system of professional training in ABA as well as delivery of behavioral services with support from national health insurance. Act no. 201/2017 Coll. (Zákon 201/2017), which amended the Act no. 96/2004 Coll. (Zákon 96/2004), was adopted shortly after this. It recognized behavior analyst, assistant behavior analyst and behavior technician as health-care professions and established conditions for their professional education and scope of practice (Act on Allied Health Professions). The Ministry of Health of the Czech Republic subsequently issued related decrees that described the minimum standards for theoretical and practical training (Vyhláška [Decree] 39/2005 Coll.) and detailed the scope of practice of each profession (Vyhláška [Decree] 55/2011 Coll.). In 2017, the new allied health profession of behavior analyst was established, including the ranks of assistant behavior analyst and behavior technician. In 2017, Jana Gandalovičová, the mother of a child on the autism spectrum and one of the main drivers of this work, was awarded Autism Speaks’ International Award (Autism Speaks, 2017) and, in 2018, she received the BACB’s Michael Hemmingway Award (BACB, 2018).

By 2021, the CSABA had over 50 full members including local behavior analysts, assistant behavior analysts, behavior technicians, and other professionals who use ABA-based procedures in their practice. Even with this expansion of ABA, intervention as well as education based on ABA principles is most often implemented in the form of individual home-based programs. This may be changing, though, as ABA-based practices have recently found their place in speech therapy in the Czech Republic. The Association of Clinical Speech-Language Pathologists (SLPs) has granted approvals to several continuing education courses of SLPs focused on ABA. There also has been increased interest from schools to use practices based on ABA. In fact, in 2021, the first medical facility providing behavioral interventions for children with neurodevelopmental disabilities was opened in Prague.

At the same time, there was a concerted effort to translate behavior analytic materials into Czech, starting with the translation of the multimedia resource SimpleSteps (2016) and the “white book” (Cooper et al., 2020). Thus, increased numbers of people with neurodevelopmental disabilities have access to interventions based on ABA (Kingsdorf & Pančocha, 2021) and there is a positive trend of increasing interest of schools in providing in-service staff training in ABA-based practices. However, most of the ABA-based services in the country are still paid out of pocket by individuals as public funding is scarce and health insurance companies refuse to recognize ABA-based interventions as essential for individuals with neurodevelopmental disabilities.

## Denmark

The situation in Denmark with regards to ABA is at an early stage. The Danish Association of Behavior Analysis (DABA) was founded in May 2003 and has 221 members. The Danish Association is driven by the demand for services among families affected by autism, but it is a small organization compared to those in most of the other Nordic countries. The Danish Association has been actively lobbying for better education for children with autism in the local and primary schools. Several professionals provide private ABA-based services for individuals with autism or other developmental disabilities in Denmark, but the educational level of these professionals is not regulated. A few professionals work in a public setting in an ABA position. As of July 4, 2022, there are only four BCBA’s in Denmark and one professional with a MSc in ABA.

The positive effect of ABA-based interventions for children with autism is recognized by the Danish government, but the endorsement varies across the country. One Danish Commune is offering ABA-based interventions as an alternative to a special school and kindergarten placement. Only few schools and institutions hire ABA professionals, and no government agencies offer full ABA-based programs. There are no academic programs in behavior analysis. The lack of university programs in ABA is one of the main obstacles that impede the development of ABA in Denmark. In addition, the strict rules regarding language create barriers that makes it difficult for many to access international training resources, most of which are offered in English. Translating the technical terms into plain professional language is going to be crucial for the future development of ABA in Denmark.

Together with the other Nordic countries (Iceland, Sweden, Norway, Finland, and Denmark), the Danish Association of Behavior Analysis is involved in developing quality assurance of behavior analysis within the Nordic context. This is of great importance for the future development of ABA in Denmark.

## France

ABA has been developing slowly in France over the last 2 decades, driven by the demand for services among families affected by autism but with little organization and coordination. Autism had long been considered a psychosis and as such autistic persons were considered objects of care rather than people who would benefit from education. In fact, only 30% of children and youth on the autism spectrum received any services, mostly provided in outpatient psychiatric hospitals (i.e., 60%; Granger-Sarrazin, 2013). According to the official journal published by the Council for Economic, Social and Environmental Affairs (Prado, 2012), there was little emphasis on mainstream inclusion and intensive behavioral interventions were available only at the economic expense of families.

On March 8, 2012, the High Authority for Health (HAS) and the Agency for the Assessment of Quality in Social and Medico-Social Establishments (ANESM) published “Good Practice Recommendations for Autism and Other Pervasive Developmental Disorders” (Recommendations de bonne pratique, 2012) that included ABA therapy categorized at scientific level Grade B. Despite this recommendation, the major issue in France remains the lack of behavior analysts and of university training courses in behavior analysis. As of December 2021, there were two BCBA-Ds, 90 BCBAs, 19 BCaBAs, and 28 active RBTs. The majority (if not all) of the BACBs are working in the field of autism and other developmental disorders and are psychologists or therapists providing the following services: supervisions/consultations for institutions and professionals already certified or seeking a certification, training in ABA to professionals and parents, parental support, contractual consultants/supervisors for other private training and supervision providers, and at times direct interventions because of the lack of qualified technicians.

At the time of this writing, there are two active VCS providers in France: Association Française-Les Professionnels de l'Analyse du Comportement / ABA Online and Association Agir et Vivre l’Autisme (BCBA and BCaBA, 4^th^ ed.). Those two associations offer several sessions a year for approximately 45 students. The only public French university that provided a master’s degree in ABA was the University of Lille, which admitted 5–15 students annually since 2003. The University of Lille was a VCS provider until 2021, but for reasons that are not known publicly, it now is listed as “retired” on the ABAI website (ABAI, 2021).

**Fig. 1:**
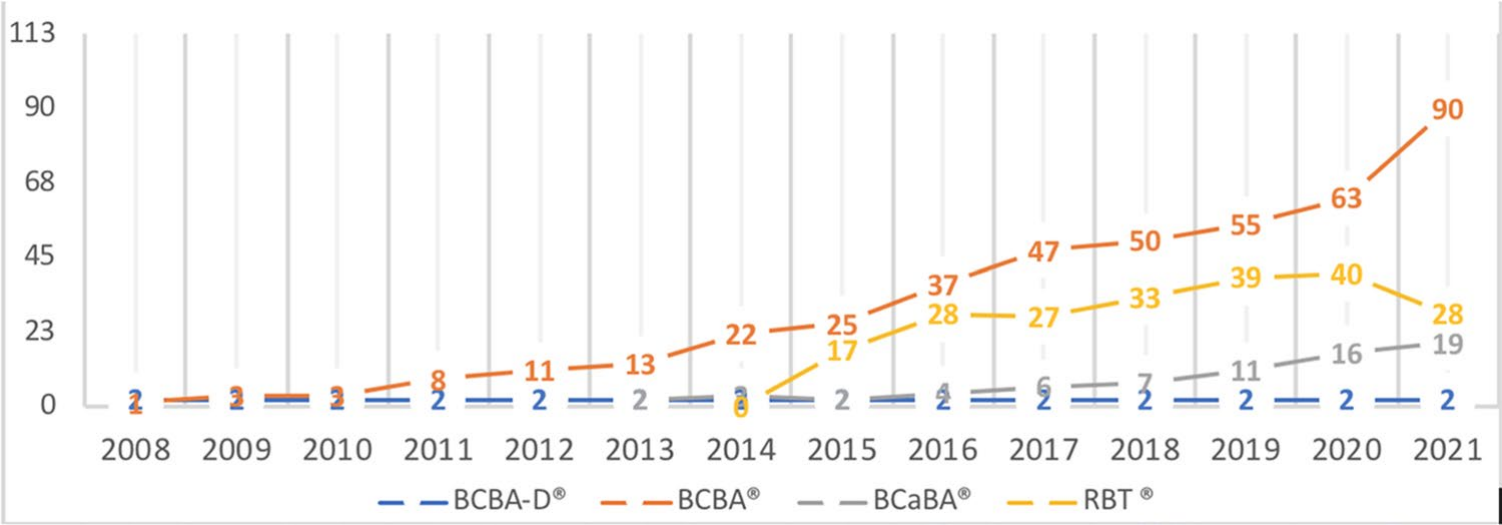
Cumulative Number of Certified Behavior Analysts in France since 2008

The strong psychoanalytic undercurrent at the French universities limits the development of university programs in ABA, and the language barrier to access and succeed in the BACB exam further impeded the development of ABA. Despite these obstacles, France is one of the five European countries with the highest number of BACB certificants. Figure 1 shows the cumulative numbers of certified professionals in France since 2008.

**Table 1:** Education of Certified and Noncertified Professionals in Spain (n = 45)

	Certified % *( n)*	Uncertified *(n)*
Age, *M ( SD), n*	38 (10), 20	40 (9), 25
Education		
High school	0% (0)	3% (1)
University, bachelor	35% (7)	31% (11)
University, doctoral	5% (1)	11% (4)
University, master	60% (12)	54% (19)
University field of study		
Behavior analysis*	20% (4)	14% (5)
Education	15% (3)	23% (8)
Speech language pathologist	5% (1)	6% (2)
Medicine	5% (1)	3% (1)
Psychology	40% (8)	46% (16)
Other	15% (3)	9% (3)
Behavior analysis training		
Non-university (short courses)	0% (0)	9% (3)
Non-university (on-the-job training)	0% (0)	23% (1)
Non-muversity (specialized course)	50% (10)	29% (10)
Uttiversity (specific courses)	15% (3)	3% (1)
University (non-degree program)	30% (6)	49% (17)
Other	15% (2)	9% (2)

(*) Master's (non-degree program) or doctontl studies with a focus *in* applied behavior analysis.

**Fig. 2:**
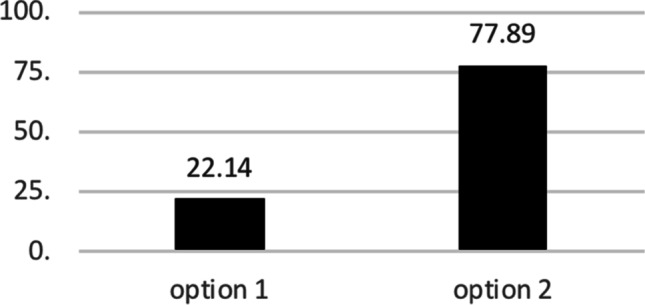
Percentage of Reference of Future Direction for French Behavior Analysts

After the BACB’s announcement related to changes in their global policy, with the guidance of Dr. Neil Martin, director of international development of the BACB, an online survey was created to gain insights into what option the French community of professionals working in ABA would prefer between maintaining certifications through the BACB or the creation of a national nonprofit organization. Of the 140 respondents, including 2 BCBA-Ds, 32 BCBAs, 9 BCaBAs, 12 RBTs, 72 noncertified VCS graduates and 13 respondents who did not identify their professional designation, 22,14% preferred to look for ways to continue accessing certification through the BACB (Option 1), whereas 77,89% preferred to work on creating a national organization (Option 2; Figure 2).

**Table 2:** Work Environment of Certified and Noncertified Professionals in Spain (n = 45)

Work setting	Certified % (*n*)	Uncertified % (*n*)
Non-for-profit	5% (1)	3% (1)
Private education center	10% (2)	17% (6)
Public education center	10% (2)	3% (1)
University	0% (0)	6% (2)
Private business	40% (8)	26% (9)
Public health service	0% (0)	6% (2)
Freelance	30% (6)	31% (11)
Other	5% (1)	9% (3)
Area of work		
Disability	5% (1)	3% (1)
Autism	85% (17)	91% (32)
Psychotherapy	0% (0)	3% (1)
Education and supervision	5% (1)	3% (1)
Other	5% (1)	0% (0)
Years of experience		
1 – 5	45% (9)	51% (18)
6 - 10	25% (5)	26% (9)
10 - 20	20% (4)	11% (4)
More than 20	0% (0)	9% (3)
Unspecified	10% (2)	3% (1)

Through a transparent and democratic process, the Organisation Nationale des Professions de l’Analyse du Comportement (National Organization of Behavior Analysis Professions; ONPAC) was officially founded in May 2020 as a nonprofit Association Loi 1901^3^. The purpose of ONPAC is to promote the practice of behavior analysis in France in an ethical framework that respects the consumers. ONPAC has currently eight active committees with over 40 volunteers working on different aspects of the future national certifications in ABA.

Given the limited number of ABA professionals and undergraduate and graduate programs in behavior analysis in France, gaining recognition of a new professional title is challenging. However, for the ONPAC, earning state recognition for its certificants and supporting the community of behavior analysts and the consumers remain the top priorities.

## Germany

The field of behavior analysis has (very) slowly been growing in Germany over the last 2 decades. It started out with one BCBA in 2004 and grew up to 52 BCBAs, one BCBA-D, and eight BCaBAs as of November 2021. The Association for Behavior Analysis Germany was founded in 2011 and has been an affiliated chapter of ABAI since 2013. Also in 2013, a certification program was launched, in cooperation with the University of North Texas, to provide coursework that enabled students to become board certified (after passing the BACB exam). Two cohorts started and most of them completed their certification process by 2019, which helped to boost the number of certified practitioners in Germany. Due to a lack of new students, the program was discontinued.

As of December 2021, ABA is not yet recognized as a science or practice by German authorities. Providers can claim funding for “autism specific therapy,” which usually gets covered but there are few standards to qualify as a service provider for people with autism. The lack of university programs, let alone ones in German, is slowing the dissemination of ABA in Germany. Despite these difficulties, many people are interested in the field through participating in workshops or conferences offered in neighboring countries to reading professional books (e.g., Bördlein, 2015; Keenan et al., 2014b) or papers.

A logical next step, therefore, is to partner up with universities (in Germany and other German-speaking countries) to offer ABA courses to students studying psychology, health services, organizational management, education, and similar fields. It is hoped that this would spark interest in students in the field of ABA, both scientifically and practically and enhance consumer protection.

## Hellenic Republic (Greece)

In the Hellenic Republic, the first public university departments of psychology were established in the early 1990s, and it is in this context that the first two university courses in the Hellenic language in experimental and applied behavior analysis were established at the Department of Psychology at the University of Crete in 1996. The program moved to the Department of Psychology at Panteion University of Social and Political Sciences (in Athens) in 2006, where it expanded to a seven-semester training sequence in experimental and applied behavior analysis, in the context of its bachelor of science degree in psychology. Behavior analytic training at Panteion is supported by a comprehensive 1,000-page Hellenic-language introductory text now in its third edition (Mellon, 2013), and supplemented by translations of three seminal books of B. F. Skinner. Students who have completed a series of three introductory courses may enroll in a laboratory course at the Laboratory of Experimental and Applied Behavior Analysis, which provides facilities for experimental research with adults, children, and pigeons. Qualified students may then begin a two-semester undergraduate research thesis in the laboratory as well as a one-semester introductory practicum in ABA at one of several specialized education centers staffed by graduates of the Panteion program, with joint supervision by university faculty.

With only two qualified behavior analytic professors in the entire National University System, the establishment of a master’s degree program is not yet feasible. However, doctoral training in the field is offered both at Panteion University and at the Department of Philosophy, Pedagogy, and Psychology at the University of Athens, which also offers introductory courses in ABA in its undergraduate and postgraduate programs in developmental psychology. Courses in behavior analysis are also offered at several private colleges. Recent recognition of degrees from qualified private institutions of higher learning as equivalent with degrees from the Ministry of Education is expected to increase the number of behavior analysts engaged in tertiary education and research, and to facilitate the establishment of master’s-level training programs.

The Hellenic Community of Behavior Analysis (ABA Greece) was established at the close of the 2010 Conference the European Association for Behaviour Analysis (EABA) in Rethymno, Crete, and incorporated at Panteion University in 2012. The Community has over 200 members and is currently organizing its Fifth Biannual Scientific Conference; over 100 Hellenic- and English-language presentations from past conferences have garnered thousands of views on the internet. ABA Greece is a National Member Organization of EABA.

In 2015, ABA Greece hosted the inaugural Summer School of the EABA. In 2017, the organization established a 600-hour introductory outreach training program in behavior analysis; in 2019, it was incorporated in the newly established Panteion University Continuing Education Center. In 2021, this accessible overview of behavioral philosophy and its basic and applied sciences, which includes a supervised introductory practicum, was authorized by the Ministry of Education to provide continuing education, with public financial support, for all interested preschool, primary, and secondary-level educators across the country. Others who undertake this training work in fields such as psychiatry, nursing, social work, and speech therapy, and many study behavior analysis to better serve the needs of their loved ones. This outreach program has done much to increase awareness of behavior analysis and to displace misconceptions about behavioral philosophy of science in the Hellenic Republic.

In the 2010s, VCSs were established in Athens at Deree College and by the Association for Training in Neurodevelopment/Monorodi day intervention center, but both programs have been retired after the BACB’s December 2019 decision to no longer certify non-U.S. based behavior analysts. According to the BACB Registry, there are 28 RBTs, seven BCaBAs, and 17 BCBAs currently working in the Hellenic Republic. Low market value due to the absence of national and European Union recognition of the BACB certification, combined with its high costs relative to local incomes, limited its pursuit among qualified behavior analysts in our country.

As an independent profession, behavior analysis is not currently recognized by government authorities, and qualified practitioners must obtain positions on the basis of their training in a related field, such as psychology, speech therapy, and vocational and special education. As is true elsewhere, a substantial majority of professional behavior analysts in the Hellenic Republic work with children and adults with developmental and learning disabilities. Qualified practitioners have established more than a dozen multidisciplinary centers providing behavior analytic service in Athens, Thessaloniki, Xalkida, and Nafplio.

Unfortunately, austerity programs beginning with the 2008 economic crisis and continuing with the COVID-19 pandemic have restricted both government and parental financial resources and, as a consequence, the expansion of organized centers for behavior analytic services. This has meant that many skilled behavior analysists provide low-cost, home-based and parallel school support services for struggling Hellenic families, without systematic organizational support. ABA Greece is currently developing a national certification mechanism.

## Hungary

Like other countries, the first steps towards the dissemination of ABA in Hungary were taken by parents of children diagnosed with autism spectrum disorder (ASD). The first ABA program was supervised by professionals from Germany and established and funded by parents in 2009. This family was joined by other families in need who wanted to provide evidence-based treatment for their children. The first Hungarian Board Certified Behavior Analyst (BCBA) received certification in the United States in 2007. At the time of this writing, there are six Hungarian BCBAs, but only four of them live and practice in the country.

Within the last 10 years, two foundations were established to further disseminate the science of behavior analysis. Through the support of national and international grants, services were made available for many children with autism and challenging behavior, as well as for mainstream schools integrating children with behaviors that challenge. Although Early Intensive Behavioral Intervention (EIBI) and the Early Start Denver Model (ESDM) are mentioned in official autism practice guidelines in Hungary as best practices, no board certified or trained ABA professionals have been involved in developing these guidelines.

Unfortunately, there still are many misconceptions about behavior analysis in Hungary, not only among professionals working with people with autism, but in university-based special education departments as well. The small number of ABA professionals who practice in Hungary have been working on dissemination over the past 10 years. This involved establishing an ABA therapy center to provide early intervention services for 20 families with children with autism, as well as reaching more than 30 schools and hundreds of people through consultations, presentations, and workshops.

The first VCS in Hungary was approved by ABAI in October 2019 at Eötvös Loránd University. With the support of the 2020 International Development Grant from ABAI, the Hungarian Association for Applied Behavior Analysis (Magyar Alkalmazott Viselkedéselemzés Egyesület; MAVEE; www.mavee.hu) was established on January 28, 2021, and an online campaign was launched to recruit applicants for the VCS. A second postgraduate behavior-analytic course sequence was established at the University of Debrecen in 2021. Both course sequences welcomed their first cohort in September 2021, which means that the country will be enriched with 18 new behavior analysts by the summer of 2023.

The newly established Association plans to focus on organizing a conference to disseminate behavior analysis and to share information about the course sequence in the summer of 2023. Efforts also are underway to explore the possibility of other universities hosting a behavior analytic course sequence. One of the projects for the first year of the Association will be recruiting as many members as possible through free online events to disseminate the science and practices of ABA to both professionals and service users.

Work has begun on translating behavior-analytic literature into Hungarian. In fact, the outcome of the first project of the Association has been completed; the translation of behavior-analytic terms into Hungarian is now free to download from the Hungarian Association of Behaviour Analysis’s website (2022). As a second project, MAVEE members established the first *Codes of Ethics* for Hungarian behavior analysts (2022b). This was achieved through successful collaboration of Hungarian BCBAs and ABA practitioners both living in the home country and abroad.

## Ireland

As in other European countries, the Irish behavior analytic community coalesced under the minimum practice standards and ethical framework provided by the BACB. Despite the lack of statutory recognition, Irish behavior analysts continued to secure positions in the education, voluntary, community, and private sectors. Their contribution to evidence-based social policy is evident in health, social care, and education policy and guidelines. Positive Behavior Support (PBS) is now on the Irish statute book, and services providing support to people at risk of challenging behavior are required to provide training in PBS (Department of Health, Ireland, 2013). In their position paper on the remediation of challenging behavior, the College of Psychiatrists of Ireland (2016) uses behavior analytic terms and names behavior analysts a necessary part of interdisciplinary teams.

Leslie and Tierney (2013) provide a detailed account of the historical development of behavior analysis in Ireland, identifying the first professional organization as the Behavioural Engineering Association, which first met in 1970. This group developed into Behaviour Analysis in Ireland (BAI), a professional body inaugurated in 1977. The BAI continued until 2013 when it became the Division of Behaviour Analysis (DBA), a division of the Psychological Society of Ireland (PSI). As a Division of the PSI, the DBA represented those with a primary degree in psychology. This led to a divergence between behavior analysts who had a primary degree in psychology and those who had primary degrees in other disciplines. At the same time, graduates from other disciplines continued to accept places on the MScABA programs in Ireland, the UK, and elsewhere. Some of these graduates returned to Ireland and secured positions in academia, clinical, and educational settings. The number of board certified behavior analysts (BCBA) in Ireland grew from 69 in 2013 to 187 in 2020. The DBA and the wider Irish behavior analytic community had discussed ways to progress professional recognition for Irish behavior analysts and the announcement by the BACB in December 2019 regarding the changes in eligibility to sit the exam, brought these discussions into sharp focus.

In February 2020, the DBA held an Extraordinary General Meeting (EGM). At the EGM, the DBA committed to facilitating the development of an independent organization that could represent the interest of all behavior analysts in Ireland. Over the following18 months, a consultation process was facilitated by members of the DBA and a working group of behavior analysts. This working group developed the governance structures required to form a company limited by guarantee (CLG). This CLG was inaugurated on October 6, 2021, and a board of directors was elected. This board plans to register the company name The Irish Society for Behaviour Analysis.

While facilitating the development of an independent organization, the DBA continued to pursue accreditation from the PSI for the MScABA programs in the Republic of Ireland (Trinity College Dublin and the National University of Ireland Galway). Both of these MSc programs were accredited by the PSI and verified as course sequence by ABAI, and accept applicants without a primary degree in psychology. Accreditation provides a route to professional recognition for those with a primary degree in psychology. However, without addressing regulation for those without primary degrees in psychology, Ireland could diverge from the strategy adopted by the UK-Society for Behaviour Analysis (UK-SBA), the Behavior Analyst Certification Board in the United States and Canada, and the likely direction of European and international standards professional development. A national divergence from international standards would restrict cross-border as well as international professional mobility (Leslie & Tierney, 2013). Permitting a situation to develop where a behavior analyst trained in Belfast is unable to apply for a position in Dublin is untenable.

The Irish Society for Behaviour Analysis (ISBA) will advocate for the professional regulation of ABA in Ireland and seek to align with international standards of practice as they develop. The principal objective of ISBA is to work to protect the public by advancing public accountability, promoting high standards of professional conduct, professional education and continuing professional development for behavior analysts in Ireland. In the past, the BACB provided shared ethical standards and a means of assessing minimum standards for competence in the practice of ABA. However, the lack of statutory recognition and regulation of ABA inhibited further growth in Ireland, and many talented behavior analysts left Ireland or the profession as a result. Now it is time to share ethical and practice standards internationally while addressing professional regulation nationally.

## Israel

**Fig. 3:**
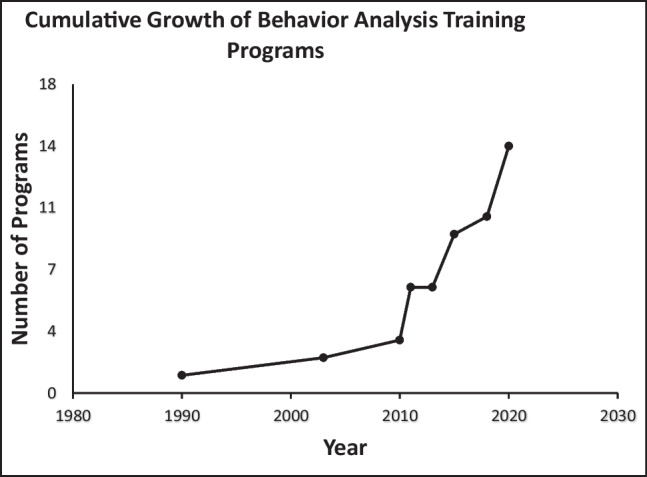
Cumulative Growth of Behavior Analysis Training Programs in Israel

The state of Israel is small (22,072 km^2^) country and includes 9.3 million people. ABA services have a relatively short history. The Israeli Association for Applied Behavior Analysis was established in 2003 and a few years later the Israeli Association for Certified Behavior analysts was founded (Ayvazo, 2015). The first formal training program in ABA launched 1990 and a rapid growth in programs has been noted since 2013. In 2021, there were 12 ABA programs residing in a number of colleges and a university as a postgraduate program and one master of arts graduate program (Figure 3). Ten of the programs are VCSs verified by the ABAI. In 2021, the BACB certificants' registry listed a total of 116 certified behavior analysts: 56 active BCaBAs, 52 BCBAs, and eight BCBA-Ds. Ayvazo (2015) estimated that in the first 2 decades of preparation of behavior analysts, more than 1000 specialists have entered the profession. By 2022, it is estimated that this number has doubled to more than 2000 professionals. Despite the rapid growth of practicing behavior analysis in Israel, there is still no formal recognition of behavior analysis as a profession in the state of Israel (Ayvazo, 2015).

The BACB's announcement of the change in terms of international certification has fostered the establishment of the state regulation committee. The committee congregated in April 2020 and has worked diligently for 18 months. The regulation committee had set three objectives: (1) preparation of an Israeli examination and certification mechanism for licensed practice in behavior analysis (the exam will be based on the current BACB task list); (2) establishment and adherence to the scientific and clinical standards set by the BACB and the ABAI, with adaptations to the Israeli laws and cultural norms as needed; and (3) defining and determining the criteria for field experience of future behavior analysts based on the standards set by the BACB and ABAI, with adaptations that consider the needs and the human resources available in Israel to provide instruction and supervision.

The regulation committee was composed of 19 members. They entailed an equal representation of the two professional organizations in Israel, representatives from academics who hold tenure-track positions at higher education undergraduate and graduate programs in Israel, and representatives of key health-related organizations for families with children on the autism spectrum. The state regulation committee included seven subcommittees: Criterion, practicum, examination, exam operation committee, ethics, certification maintenance, and governance coordinating committee. Each subcommittee had its defined goals and working plan that was presented and discussed within the plenum. The regulation committee commenced in December 2021 and the chairs of the two professional organizations for behavior analysts in Israel, who also led the state regulation committee, began spearheading a process of uniting into a single representative organization. To date, Israeli behavior analysts successfully accomplished the establishment of a new, united professional organization for behavior analysis in Israel. This organization is charged on executing the regulation committee recommendations at the state level as well as applying for international grants to support the national process.

## Italy

The word behavior analysis (and behavior modification) became known among a small group of Italian psychologists in the early 1970s, thanks to a Conference at Villa Falconieri in Frascati (Rome) attended by Fred S. Keller, among others (Moderato & Presti, 2006, 2019). By attending some other international meetings, like the Experimental Analysis of Behavior Group (EABG) meetings in Liege in 1983 and 1988, and the series International Congress on Behavior Studies, in particular the inaugural one in Guadalajara, Mexico, in 1992, a small group of pioneers had the chance to meet scholars such as B. F. Skinner, Fred S. Keller, William N. Schoenfield, Charles A. Catania, and Peter Harzem. The contextualistic soul, together with the particular interest in the developmental behavior analysis that characterizes this pioneering group of behavior analysts, can be traced back to the contribution of Sidney W. Bijou (1984, 1993).

The Second International Congress on Behavior Studies, which took place in Palermo in 1994, offered a larger Italian community the chance to meet the most influential scholars of the international behavior analytic community, including Fred S. Keller. At that meeting, he delivered his last speech before his death. It should be noted that the first International Conference of ABAI took place in Venice in 2001, at which the first nucleus of the European Association for Behavior Analysis (EABA) was established, followed by the official founding of EABA that took place in Parma 2 years later (Arntzen et al., 2009). Thus, though small, the Italian behavior analytic community played a significant role in the European development of behavior analysis.

Behavior analysis is not officially taught in any Italian university. There is no professor of behavior analysis, and there is no master’s degree (i.e., Laurea Magistrale) program in behavior analysis. Some private institutions deliver postgraduate course sequences in ABA of different quality and length, at three levels (technician, assistant, analyst) that resemble the BACB levels. The term “certification” has no status in the Italian welfare system because health professions (e.g., physicians—including child psychiatrists—psychologists, speech pathologists, physiotherapists) are ruled by state laws, whereas the Ministry of Education regulates special educators. As in other European countries, behavior analysts are not formally recognized or regulated as a distinct profession in Italy; the profile of behavior analysts is not (and perhaps cannot be) clearly defined because, in many respects, it overlaps with some functions and task lists of the psychologists.

In 2011, the Higher Institute of Health (l’Istituto Superiore di Sanità; ISS) published Guideline 21 (LG 21). ISS is a public law body that, as the technical-scientific body of the National Health Service in Italy under the Ministry of Health, carries out research, experimentation, control, consulting, documentation, and training in public health. LG 21 acknowledges ABA-based procedures as evidence-supported treatment (EST). Autistic children are the main client group of child psychiatry services. It is sad to report, but few child psychiatrists are trained in behavior analysis and have the minimum knowledge to understand basic principles of behavior analysis without bias or misrepresentations (90% are psychoanalytically trained). For this reason, ISS funded and organized a course sequence for them not to become behavior analysts but to make them able to distinguish whether an intervention appointed as ABA-based is well-designed, well-implemented, and effective. The third edition of this course sequence is about to start. The instructors are selected behavior analysts.

In 2014, the Italian Society of Experimental and Applied Behavior Analysis (SIACSA, 2014) was funded. The SIACSA established a directory of behavior analysts (AdC), assistant behavior analysts (aAdC), and technicians (Tac) that meet minimum professional standards. The Associazione Tecnici ABA (ASSOTABA, 2014) is an association that primarily includes behavior technicians. In February 2020, a few days before the lockdown for COVID-19, SIACSA and ASSOTABA merged to create the new association ABA-Italia (https://www.abaitalia.org/), which includes 355 behavior analysts (AdC), 155 assistant behavior analysts (aAdC), and 456 behavior technicians (TAC).

The two leading family associations in the field of autism, Associazione Nazionale Genitori Soggetti Autistici (ANGSA, 2020) and Associazione Nazionale Famiglie di Persone con Disabilità Intellettiva e/o Relazionale (ANffAS, 2020), endorsed ABA Italia. ANGSA and ANffAS play a control role chairing the ABA Italia Ethics Committee. Finally, ABA Italia, through its Scientific Committee, which includes the most distinguished Italian behavior analysts, developed a set of rules to define the highest quality behavior analyst training. Those rules reflect BACB criteria with some adjustments. The training for technician level is a postbachelor degree course sequence of 70 hr (instead of 40 hr for RBTs); there are two course sequences for students who want to become behavior analysts, one for early interventions and one for those who work with adolescents and young adults. This population has special needs, including quality of life, independent life, and psychological disorders like anxiety, depression, and obsessive-compulsive disorders (Oppo et al., 2019).

With regards to autism services, a child psychiatrist delivers an early preliminary diagnosis and eventually prescribes the intervention, usually consisting of 45 min twice a week with a speech pathologist and psychomotricist (a kind of developmental physiotherapist). The National Health Service does not officially acknowledge BCBA, BCaBA, RBT certifications.

## Malta

ABA is a young field in Malta with only a handful of practitioners in 2021. In April 2021, a survey was carried out within the behavior analysis community in Malta to get a clear idea of the current context. There were 14 respondents. The first BCBA achieved certification in November 2017 after completing the MSc with Cardiff University, Wales. There were two BCBAs working on the island, two RBTs, and five individuals who have completed their MScABA and are working towards BCBA certification. In December 2021, there was a significant increase and there now are nine BCBAs and eight RBTs practicing on the island, a promising progression for Malta. The University of Malta does not currently offer a VCS and all those who have completed an MSc have done so via online courses or have traveled to study and returned to the island.

Practitioners support families either on a freelance basis, or through their private clinics and practices and receive supervision from overseas. At the time of this writing, there is one ABA center in Malta with an in-house BCBA that offers government-funded projects to support children on the autism spectrum. Apart from these individual projects, ABA is not funded or supported on a general basis by the Maltese government. All respondents to the survey work to support children on the autism spectrum and their families, work in school settings, offer parent training and support, and the majority have been working in the field for fewer than 6 years.

The appreciation for ABA is slowly growing in Malta with referrals to ABA-based intervention being made by more professionals in recent months. The dissemination of the field among other professionals such as doctors, psychiatrists, speech, and occupational therapists is important because in Malta they typically are the first point of contact when families seek support.

In 2016, a talk about ABA was given during a conference and since then ABA has been slowly making its mark on the island. This, however, is only the beginning and dissemination of ABA needs to grow more rapidly and to a high quality and standard. Working with government entities and policy makers to recognize the importance of their support is a current and ongoing journey for stakeholders in the field. It is important for Malta that an entity exists to regulate the field and that practitioners are working towards certification under a regulatory body to ensure that the standard of practice remains high, as have been the expectations under the BACB.

## The Netherlands

There is no formally accredited training and/or education for behavior analysts in the Netherlands, yet a substantial number of services that use principles of ABA to support young people on the autism spectrum and their families have been founded over the last 25 years. Many of these services were set up by parent initiatives, e.g., Robertshuis (2006), de Droomboom (2006), and Stichting Raeger (2011). Using the principles of ABA, these services support children, adolescents and (young) adults with a diagnosis of autistic, developmental disorders and/or other psychiatric or behavioral diagnoses, such as ADHD, attachment disorder, oppositional defiant disorder (ODD), obsessive compulsive disorder (OCD), incontinence, and eating problems. ABA-based support is typically provided in multidisciplinary teams together with speech and language therapy as well as occupational therapy.

In a first attempt to draw up the history of ABA in the Netherlands, four activities were initiated at the beginning of 2021:

- an internet search for ABA services in the Netherlands, which yielded 40 organizations;
- a survey that was sent to all these organizations to acquire information on structural characteristics of their organization (e.g., number of staff with and without formal (ABA) training, level of educational background of staff (e.g., vocational, higher education); number and characteristics of clients, number of service locations, and type of ABA services;
- a search in the certificants’ registry of the Behavior Analyst Certification Board (BACB) for Dutch certified/registered professionals (e.g., RBT, BCaBA, BCBA, and BCBA-D); and
- archival research, in which any reference to ABA in the Netherlands is extracted from (in)formal documents in education, services, and/or personal archives.

**Fig. 4:**
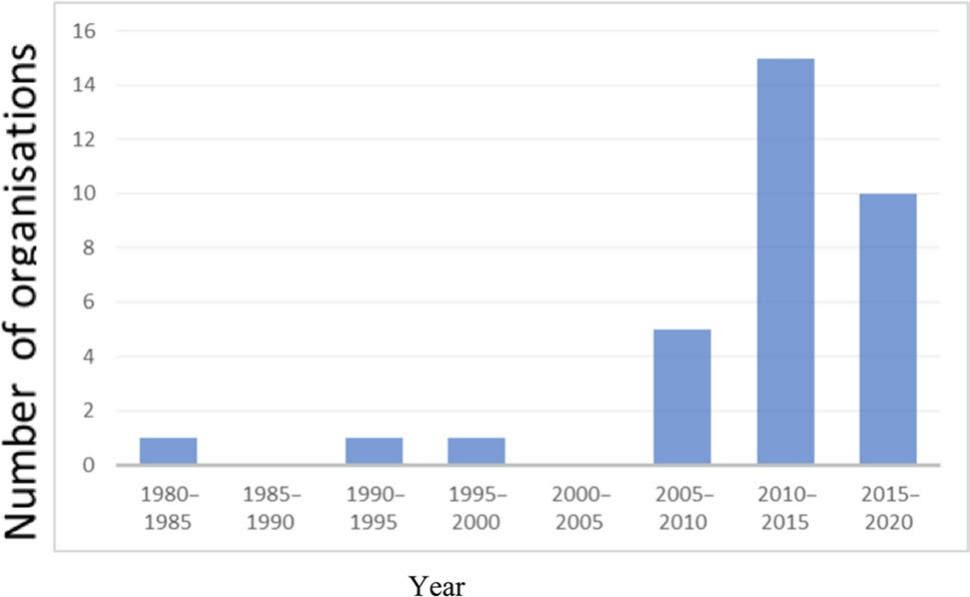
Year of Foundation of Services in the Netherlands Providing ABA

The (preliminary) results of activities 1–3 illustrate the relatively young history of ABA in the Netherlands. The first services claiming to provide ABA services were founded in the 1980s; however, the majority of organizations in operation have been set up since 2010 (Figure 4). The number of certificants has increased steadily since and in 2021 there are a total of 40. Based on the initial results of the survey (activity 2), staffing of 10/40 of the organizations ranged from 1 member of staff to 1,915 staff members (average 303 staff, *SD* = 645.5). The number of clients who received ABA-based services from these agencies ranged from 4 to 7,485 clients (average 941, *SD* = 2,362.4).

**Fig. 5:**
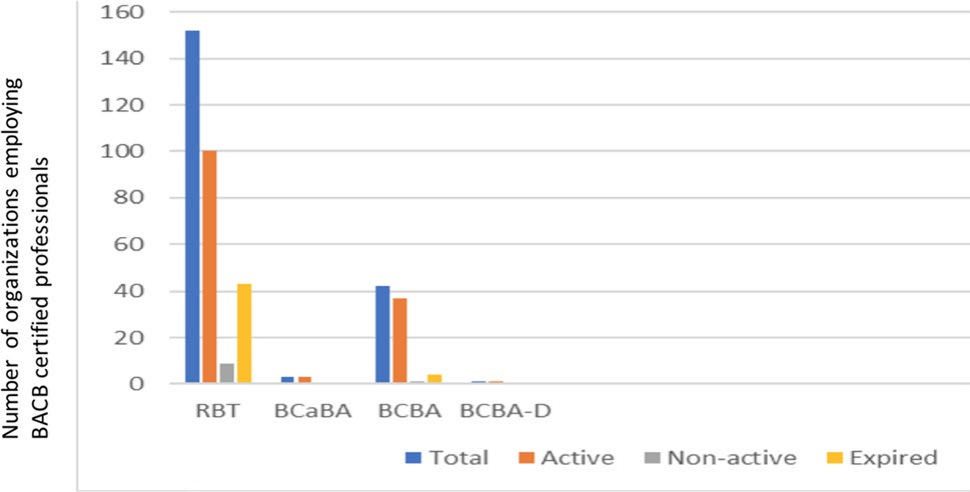
Number of BACB Certificants in the Netherlands According to BACB Registry (Reference Date Octo er 26, 2021)

**Fig. 6:**
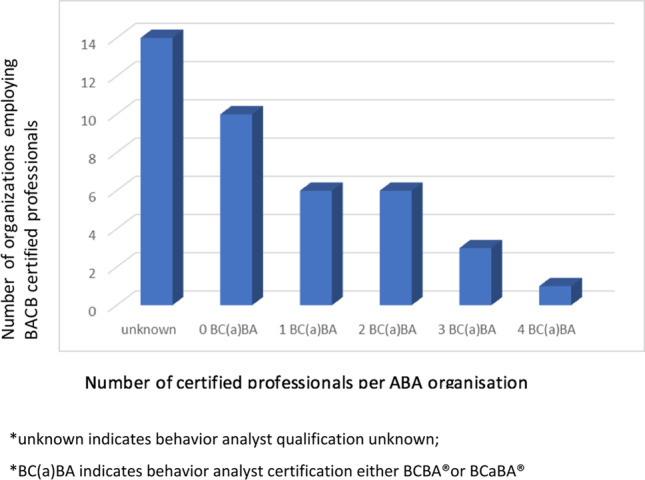
Number of Dutch ABA Organizations Who Employed Behavior Analysts

Figure 5 illustrates the number of staff with formal training and certification in ABA. In 2021, there were at total of 198 professionals who had obtained some form of registration/certification with the BACB (152 RBTs, 3 BCaBAs, 42 BCBAs, and 1 BCBA-D). Most of these staff are registered as “active”; however, 43 of the RBT registrations had expired and 3 RBTs were nonactive. Figure 6 shows that the distribution of behavior analysts employed in the ABA organizations is uneven. The majority of ABA-based organizations (16/40) in the Netherlands, employ at least one BC(a)BA; in 14 ABA organizations the number of certified behavior analysts is unknown; and 10 ABA organizations do not employ a single certified behavior analyst.

The discrepancy between ABA services has implications for service quality and intervention fidelity (Huskens et al., 2015). Neidt and Schenk (2012) explored early intensive behavior interventions, including pivotal response training (PRT), discrete trial teaching (DTT), and picture communication exchange programs (PECS), at three organizations in the Netherlands, and found that rather than offering high-fidelity ABA-based supports, they provided a rather eclectic combination of interventions.

At the time of this writing, only a handful of in-service workshops and courses in ABA are offered in the Netherlands that are not accredited by any independent accreditation bodies, such as the Dutch-Flemish Accreditation Organisation (NVAO) and/or professional bodies such as the Dutch Institute for Psychologists (NIP) or the Dutch Association for Educationalists and Pedagogues (NVO).

In order to pave the way ahead, an ABA consortium met in 2021 to emphasize the importance of the development of nationally recognized and accredited ABA training and professionalization opportunities to meet the current and future needs of ABA service users. The aims they identified were to encourage and/or maintain high standards in ABA-based support and early behavioral intervention services; to enable future accredited ABA education and training opportunities; and to secure the future of high-quality ABA-based supports in the Netherlands.

## Poland

The interest in ABA in Poland has been significant for over 20 years; as of 2021, there are 22 BACB certificants, one BCBA-D, and one BCaBA. Since the 1990s, there have been many facilities that implemented ABA-based intervention for children and adults on the autism spectrum. At the time of this writing, in every major city in Poland there are kindergartens, schools, or special centers that use the ABA principles in their pedagogy. Due to such great interest, two associations were established in Poland that are affiliated chapters of ABAI:

The Polish Association of Behavioral Therapy (PABT) was established in 2002. PABT is a countrywide nonprofit organization of active practitioners of behavioral therapy. PABT’s mission is to provide people with disabilities, in particular those with emotional and developmental disorders, and their families with multidirectional, comprehensive, and professional help. PATB’s main aim is promoting behavioral therapy among therapists and academics. At the time of this writing, PABT has more than 44 registered members from every significant behavioral therapy center in Poland, as well as parents of persons undergoing behavioral therapy and other concerned experts (physicians, academics, and students). It cooperates with a number of Polish centers and institutions focused on providing effective help to persons with autism and publishes the informational periodical *Krok za krokiem (Step by Step),* which contains articles by world-renowned experts on teaching persons with autism. PABT has promoted behavioral therapy by organizing specialist trainings for a wide range of attendees (mainly the teachers and parents of the emotionally and developmentally disabled). Over 1,000 participants attended such trainings in 2020.

The Polish Society for Behavioral Psychology (PSBP) was founded in 2002 at the Institute of Psychology of the Jagiellonian University (JU), Poland. Members of PSBP are not only psychologists and psychology students, but also representatives of related disciplines, mainly educationalists and therapists. The main objectives of PSBP are to develop and promote psychological knowledge, with special focus on behavior analysis as the science of behavior and the environmental factors that influence behavior and to propagate the idea of effective behavioral therapy as a form of psychological intervention and to disseminate knowledge about therapeutic methods and their effectiveness. PSBP strive to achieve the above goals through science, publishing, and training activity as well as through work for the community of psychologists and psychotherapists.

Postgraduate studies in ABA are conducted at universities in several larger cities in Poland. University of Social Sciences and Humanities conduct studies in Warsaw, Poznań, Katowice, Sopot, and University of Gdansk in Gdańsk. Two verified courses sequences have been established: a course run by University of Social Sciences and Humanities in Warsaw applied behavior analysis, “Working with Individuals with Developmental and Behavioral Disorders,” Monika Suchowierska-Stephany, PhD, BCBA-D, BACB VCS coordinator; and a course run by University of Gdansk Applied Behavior Analysis: “Teaching Children and Youth with Autism Spectrum Disorder.” The course started in 2015. Since then, 66 students have completed the course. The third edition has finished in February 2021. Anna Budzinska, PhD, was the BACB VCS coordinator.

There are three certification systems for teachers and other specialists in Poland: The Polish Behavioral Therapist License was established in 2010. The boards of two Polish chapters of ABAI (i.e., PABT and Polish Society of Behavioral Psychology) have been working on a joint program—Polish License of Behavioral Therapist—to standardize formal requirements for behavior therapists in Poland. The purpose of these activities is to consolidate and formalize professional training for behavioral therapists in Poland, and thus define the requirements for practicing therapists to make behavioral therapy clearly identifiable by the highest quality of therapeutic services. The Polish License of Behavioral Therapist runs an integrated system of theoretical and practical training to enable trainees to gain the title of behavioral therapist. At present, 229 people in Poland have been granted the title of licensed behavioral therapist, 31 people have the title of behavioral supervisor, and 4,000 are in the process of acquiring qualifications.

“Teacher of a Small Child with Autism” courses are run by the Institute for Child Development (IWRD) Teacher Training Center since 2012. So far, 399 people have been awarded the certificate of “Teacher of a Small Child with Autism.” The IWRD was created through the initiative of the Foundation-Institute for Child Development in 2006. The IWRD is the first and the only institution in Poland that is fully modeled on the Princeton Child Development Institute model created by Krantz and McClannahan. The mission of the IWRD is to provide comprehensive assistance to children with autism to help them achieve the highest possible level of independence. In 2010, the Teacher Training Center was created in the IWRD. IWRD trainers share experience and knowledge by holding ABA training sessions for teachers, psychologists, pediatricians, and students from all over Poland and abroad. The IWRD Teacher Training Center is accredited by the school superintendent; 7,000 people participated in different training courses over last 11 years. The IWRD Teacher Training Center offers courses about use of ABA principles when supporting children with ASD. This is a three-step course: “ABA in Intervention for Children with ASD” is a basic course on this topic that was created by Dr. Anna Budzińska, the director of the IWRD. The “Teacher of a Small Child with Autism” certificate involves a practical internship, a continuation of the theoretical three-step training program in behavioral therapy for small children with ASD.

The Behavior Analyst Certificate (CAZ) was developed in 2021 by the Institute for Child Development in Gdansk and the University of Gdansk. It is the first certificate in Poland that is fully based on postgraduate studies organized at universities. A person applying for the certificate must complete postgraduate ABA studies, 1,000 hours of practice under supervision, and pass the final practical and theoretical exams. High requirements related to obtaining the certificate ensure the education of specialists in Poland who will conduct ABA interventions at the highest level.

## Spain

As of 2021, there are 35 BACB certificants in Spain (31 BCBAs, 1 BCaBA, 3 RBTs), most of them certified within in the last few years, which suggests that the number of certificants is finally gaining momentum. Twenty-six out of the 33 BCBA/BCaBAs (81%) in Spain are graduates from ABA España’s program. The current rate at which new candidates are becoming certified has increased by a factor of 2.5 over the last 5 years. The rate of certification lapses calculated as the number of inactive BCBA and BCaBA certificants divided by the number of active and inactive certificants is 3.4%, which suggests that certificants see value in preserving their certification after it has been acquired. In addition, Spanish nationals and residents have had a high pass rate in certification exams.

The data generated through a survey conducted in 2019 by the Spanish ABAI chapter ABA España suggests that certified and uncertified professionals do not differ in their training and sociodemographic characteristics (Tables 1 and 2). This observation is consistent with the fact that only about one third of ABA España’s students from Spain end up becoming certified. The limited interest in professional certification may be due to the fact that private professional certifications in the EU market are voluntary and useful only for their perceived prestige and acceptability among stakeholders.^4^ Private professional certifications cannot be written into law unless an EU-wide scheme is developed, which seems out of reach in the current environment. With the recent change in international focus of the BACB it is possible that certification and to some extent training will slow down over the next few years.

In general, ABA services are not covered by the public or private insurance sectors in Spain. However, ABA service providers can be subcontracted by the public health system on a case-by-case basis to serve families who have been granted allowances as per the law for services to dependent individuals.^5^ A similar arrangement is possible for professionals who become registered as subcontractors of regional health services for people with autism and other disabilities.^6^ Apart from these mechanisms, families have to cover the cost of behavioral services themselves. In spite of these challenges, the professional field continues to grow with new applied programs being created every year.

## Sweden

The history of behavior analysis in Sweden can be traced back to 1951 when B. F. Skinner gave a presentation at the 13th International Conference of Psychology in Stockholm (Arntzen & Pellón, 2021). EIBI was introduced in Sweden approximately 35 years ago. However, it was not until 2004, after parental lobbying, that the first graduate-level BACB VCS in behavior analysis with a focus on autism was launched at the Karolinska Institute (Roll-Pettersson & Ala'i-Rosales, 2009; Roll-Pettersson et al., 2010). This was made possible through international support and collaboration with BACB and Shahla Ala'i Rosales, University of North Texas. Participating students were, and still are, active practitioners with academic backgrounds in education, speech-language therapy, special education, psychology, social work, and occupational therapy. In 2006, the course sequence was moved to the Stockholm Institute of Education, and in 2010, to Stockholm University, where it is currently delivered jointly by the Department of Psychology and the Department of Special Education. The VCS is ABAI approved for the fourth edition BACB task list. As of 2017, Stockholm University offers the only master’s program within Sweden in ABA. In December 2021, there were 15 active Swedish BCBAs and one BCBA-D; of these certificants, eight are psychologists, seven speech-language-pathologists and one special education teacher. In addition, more than 200 students have completed VCS coursework without pursuing certification.

In Sweden, ABA remains largely unknown to the general public. However, the embrace of behavior analytic interventions in certain preschools has increased, especially in schools that have children on the autism spectrum. Indeed, since early 2000s habilitation recommendations state that first-choice interventions for preschool children with autism should be based on the principles and procedures of ABA (Bromark & Granat, 2012). Behavior analytic supports and services including early intensive interventions are covered through taxpayer monies. In current job announcements, a number of employers from habilitation centers, psychiatric services, schools, special schools, residential and group homes request that applicants be educated in ABA.

Uppsala University is developing coursework in ABA and is also finalizing a Schoolwide Positive Behavioral Intervention and Support project, which has led to a cultural adaptation called inclusive behavioral support in school (IBIS). The IBIS project has recently obtained an external grant to evaluate this adapted version involving approximately 100 schools. At Linnaeus University and Stockholm University two doctoral projects are currently studying the PAX Good Behavior Game (GBG). After a cultural adaptation to the Swedish school context, the doctoral student at Linnaeus University is coordinating a randomized controlled trial (RCT) and the doctoral student at Stockholm University is focusing on the PAX good behavior game (GBG) with students with special education needs in mainstream settings. In addition, the PAX GBG has now been taught to more than 1,000 teachers in approximately 10% of the municipalities within Sweden.

The Swedish Association for Behavior Analysis (SWABA) was founded in 1996, and now has approximately 200 members of various professional backgrounds. Since 2020, after the BACB's decision to change its certification requirements, SWABA is involved in a collaboration between representatives from the Nordic countries (Denmark, Finland, Iceland, Norway, Sweden), discussing quality assurance of behavior analysis within a Nordic context.

In sum, though ABA appears to be becoming increasingly established and accepted on both grassroot and institutional levels in Sweden, neither the profession per se or the field is officially recognized. There are no formal requirements for staff at any level to have knowledge and/or competence in ABA. It is clear that in order to avoid low quality interventions, and potentially damage the reputation of the field, ensuring high levels of competence among ABA practitioners in Sweden is vital.

## Switzerland

ABA Switzerland was founded in 2011 and is the ABAI affiliated chapter in Switzerland. The majority of BCBAs in the country are members. ABA Switzerland actively collaborates with organizations in neighboring countries to work towards a future for behavior analysis in Switzerland.

In 2021, there are 18 BCBAs and three RBTS working in Switzerland. In the past 2 decades many students have been trained within ABA early intervention projects and are now carrying their experience further into other areas of work. Current ABA providers in Switzerland work almost exclusively in autism. There are two public institutions and one private center offering EIBI and ABA-based services. A hospital in Lausanne (CHUV) offers evidence-based services to children on the autism spectrum, under supervision of a French BCBA.

There are no university-approved courses in ABA and no VCS. There are some behavior analytic courses taught by BCBAs or ABA-trained people at different institutions as well as some RBT courses. In September 2021, a graduate level course on EIBI taught by Early Start Denver Model (ESDM) and ABA professionals was started as a collaboration between universities in Zurich, Geneva, and Ticino. There is an ABA-trained lecturer teaching ABA to future special education teachers in Fribourg.

Two studies have been published in the field of behavior analysis in Switzerland. First, a study of the implementation of EIBI for children on the autism spectrum in Switzerland (Studer et al., 2017) and second, a study of “Predictors of Treatment Outcome in Preschoolers with Autism Spectrum Disorder: An Observational Study in the Greater Geneva Area, Switzerland” (Robain et al., 2020).

Behavior analysis is not recognized as a profession yet. Billing is not possible for behavior analysts who are either privately paid or who bill through other credentials (e.g., psychology, speech therapy). There are several early intervention centers for autism approved by the Swiss government, however, only two of them are based on ABA. ABA still faces a lot of prejudice and negative sentiment in the general public and among professionals from other disciplines.

## Ukraine

Behavior analysis began to develop thanks to interested parents who organized courses in ABA, inviting the Israeli BCBA specialist Julia Ertz to teach a certified BCBA exam preparation program in Ukraine. The first graduation of specialists took place in 2012. Later, on the territory of Ukraine, training was conducted under the certified full-time training program three more times. Also, a large number of Ukrainian specialists were trained in certified training programs for the BCaBA exam in a distance-learning format. However, approximately only 20% completed their studies and among them even fewer practice. All practicing behavioral specialists are outside the scope of formal practice and work in private. It should be noted that these programs are not included in any training course of higher educational institutions and in the register of the profession of Ukraine there is no profession of a behavior analyst or a behavioral instructor. However, since 2014, behavioral therapy has been included in the roadmap for the habilitation of individuals on the autism spectrum of the Ministry of Health of Ukraine.

In 2015, leading behavior analysts in Ukraine founded the Ukrainian Association of Behavior Analysts (UABA), a nonprofit organization. In 2016, the first international conference of behavior analysis was held in Ukraine. From 2015 to 2021, several workshops, webinars and two Ukrainian conferences on behavior analysis were held. In 2019, the first two BCBAs appeared in Ukraine and became part of the UABA board.

When BACB announced that they were going to discontinue access to certification to non-U.S. residents, they offered support to other countries in their efforts to develop a national system of certification. After detailed consultation with Dr. Neil Martin (director of internationalization, BACB), the UABA worked out the qualifications and levels of education of specialists, and also adopted the *Code of Ethics* in accordance with the legislation of Ukraine.

UABA has 45 members and plans to continue to expand and conduct monthly thematic webinars for a wide range of listeners and specialists. In early 2022, there were about 10 ABA centers in Ukraine. UABA hopes to support the development of ABA in Ukraine and plans to be included in the professional community of Europe.

## United Kingdom (UK)

Until 2020, the position of behavior analysts was not strictly defined in the UK. The job title was not protected or regulated. The title BCBA was used in place of adequate national registration, in fact, only a small number of jobs advertised in the UK listed BCBA or MScABA or ABA tutor as essential or desirable criteria. There are five master’s level VCSs being taught at UK universities (ABAI, 2021). A number of universities and private providers also offer RBT training, either in person or online.^7^ The qualifications achieved in these courses remain academic qualifications that have been established to meet the BACB criteria. However, they never were officially recognized by UK government agencies; the Health Care Professional Council (HCPC) or the Professional Standards Authority (PSA, 2014)^8^. In 2009, a meeting took place at HCPC headquarters with representatives from three of the UK nations (England, Wales, and Northern Ireland). An application was prepared for recognition of the profession of behavior analysts by the HCPC, however, before this application could be submitted, in 2010 the UK government pulled the funding for any new aspirant professions. As a consequence, no further applications for recognition of new professions were accepted by the HCPC and the application for behavior analysts ground to a halt. In 2014, the UK government had introduced a new system of professional recognition via the PSA. The PSA reviews the work of the regulators of health and care professionals; accredits organizations that register health and care practitioners in unregulated occupations; and gives policy advice to ministers and others and encouraging research to improve regulation. Thus, to be accredited by the PSA, behavior analysts needed to be registered.

In 2013, UK Society of Behaviour Analysis (UK-SBA) was established. When, in December 2019, the BACB announced the termination of eligibility to sit the exam for non-U.S./Canada residents by end of 2022, the UK-SBA applied for a 3-year extension. The extension was granted and ends in 2025. In 2020, UK Society of Behaviour Analysis (UK-SBA) set up a voluntary register for behavior analysts with the aim to have this register accredited by the PSA. As of December 1, 2021, a total of 302 behavior analysts are registered on this register. Applicants for the register need to evidence that they have sufficient training in behavior analysis, they do not have to be BCBAs. The applications are reviewed and decisions are made by the UK-SBA board. In late November 2020, the UK-SBA applied for accreditation of this voluntary register by the PSA. At the time of writing, the PSA has not yet published their decision. In 2022 (backdated to 2020), the UK-SBA was added to Her Majesties Revenue and Customs (HMRC) list of approved professional organizations and learned societies. This step in the right direction means that members can claim tax relief on membership fees.

In the meantime, the UK-SBA has set up a taskforce to develop a credentialing system for behavior analysts. The target for this taskforce is to “ensure that UK-SBA works to establish over the longer-term, a framework for the professional recognition and certification of behavior analysts across the breadth of behavior analytical practice” and to “ensure that the UK-SBA meets the education and training requirements of the PSA standards and of the UK-SBA strategic plan.”^9^

## Summary

Professional regulation and registration of practice is important for behavior analysts in Europe for many reasons, including the protection of populations served, the strengthening of higher education programs, to facilitate staff mobility across Europe, and for the growth of the discipline. Although there are records of national progress of behavior analysis in some countries (Ala’i-Rosales et al., 2010; Hughes & Shook, 2007; Keenan et al., 2014a; Kelly et al., 2018), these mainly focus on dissemination of ABA rather than professional regulation. In terms of professional recognition, most European countries have used the standards and curricula set by the BACB as a starting point (Arntzen & Pellón, 2021). However, it is important for readers to understand that BACB standards did not equate to professional regulation in European jurisdictions. The certifications provided by the BACB were not nationally recognized qualifications in any European country, even prior to the decision by the BACB to change their global focus. In fact, no U.S.-based behavior analyst title, acronym, or qualification is recognized in non-U.S. jurisdictions simply because each country can only legislate for their own affairs, and this includes official recognition of professions. As in any other professions, mutual agreements that enable staff mobility can be accomplished only by countries in which behavior analysts are legally professionally recognized.

Some behavior analysts in Europe had realized this and had begun the process of seeking national recognition for the profession in their countries, whereas others were jolted into action by the BACB decision. It is not surprising that the process of professional recognition is progressing at different speeds in each country. Once behavior analysis is a regulated profession in different countries, the next step is mutual recognition across Europe. This requires professions first to be regulated nationally and second, for the qualifications to be based on harmonized minimum training requirements and recognition of evidence of training and professional experience. The EuroBA project aims to facilitate this process by providing common standards and agreed competences. As a starting point, this article offers a snapshot in time of the situation of professional recognition of behavior analysts in 21 European countries, thus providing necessary information to help move things forward and to encourage others to take up the mantle in their own country. This is important to ensure that behavior analysts are not only knowledgeable about the conceptual, scientific, technical, and ethical basis of ABA, but also have the necessary interpersonal skills to protect the science against misrepresentation (Keenan & Dillenburger, 2018) and build productive and compassionate relationships with service users (Taylor et al., 2019).

## Data Availability

The datasets generated during and/or analyzed during the current study are available from the corresponding author on reasonable request.

## Footnotes

1 Another reasons for this was the fact that the translations of the exams, websites, and all other resources were resource intensive and it was logistically not feasible for BACB to continue with translations. Extensions were granted to Australia and the UK.

2 The use of the term “autism” is culturally determined. Although some people prefer identity-first language (e.g., “autistic child”) others prefer person-first usage (e.g., “child on the autism spectrum”; Benevides et al., 2020; Kenny et al., 2015). In this article, various terms are used interchangeably.

3 An Association Loi 1901 is an agreement whereby two or more persons permanently pool their knowledge or activity for a purpose other than to share profits. cf. https://www.legifrance.gouv.fr/loda/id/LEGITEXT000006069570/

4 Private professional certifications in Spain cannot be written into law due to the European Union’s antitrust rules as they relate to professional services. In essence, individual EU members cannot endorse a professional certification because that would harm the ability of professionals from other member states to compete in the EU market.

5 Ley de promoción de la autonomía personal en personas en situación de dependencia, full text available at https://www.boe.es/eli/es/l/2006/12/14/39/con

6 This may vary across regions as Spain—competences in health and education are to a large extent transferred to the 17 regional governments in the country.

7 https://uk-sba.org/about-behavior-analysis/university-training/

8 https://www.professionalstandards.org.uk/home

9 https://uk-sba.org
