# Correction: Professional Development of Behavior Analysts in Europe: A Snapshot for 21 Countries

**DOI:** 10.1007/s40617-022-00760-2

**Published:** 2022-11-28

**Authors:** Mickey Keenan, Karola Dillenburger, Marie-Hélène Konrad, Natacha Debetencourt, Rea Vuksan, Lefki Kourea, Karel Pancocha, Sheri Kingsdorf, Henriette Juul Brandtberg, Nursel Ozkan, Helene Abdelnour, Magali Da Costa-Meranda, Steffi Schuldt, Robert Mellon, Alexandra Herman, Alan Tennyson, Shiri Ayvazo, Paolo Moderato, Natasha Attard, Jacqueline Schenk, Anna Budzinska, Javier Virues-Ortega, Lise Roll-Pettersson, Dag Strömberg, Silja Wirth, Charlotte Escané, Erika Glaus-Stuessi, Alla Moskalets, Stephen Gallagher

**Affiliations:** 1 Ulster University, Coleraine, UK; 2 Queen’s University Belfast, Belfast, UK; 3 PCM, Vienna, Austria; 4 Univeristy of Nicosia, Nicosia, Cyprus; 5 Masaryk University, Brno, Czech Republic; 6 Danish Association for Behaviour Analysis, Copenhagen, Denmark; 7 ONPAC-Organisation Nationale des Professions de l’Analyse du Comportement, M, France; 8 Panteion University of Social and Political Sciences, Athens, Greece; 9 Association for Behaviour Analysis Hungary, Budapest, Hungary; 10 Irish Association for Behaviour Analysis, Dublin, Ireland; 11 Kinneret Academic College, David Yellin Academic College, Jerusalem, Israel; 12 IESCUM, Parma, Italy; 13 Erasmus University Rotterdam, Rotterdam, Netherlands; 14 Institute for Child Development (IWRD), Gdańsk, Poland; 15 ABA Spain, Barcelona, Spain; 16 University of Stockholm, Stockholm, Sweden; 17 ABA Switzerland, Gossau, Switzerland; 18 Ukrainian Association of Behavior Analysts, Mariupol, Ukraine

**Correction: Behavior Analysis in Practice**

**https://doi.org/10.1007/s40617-022-00754-0**

This article was updated to correct the affiliations.

